# The uncharacterized transcript *KIAA0930* confers a cachexic phenotype on cancer cells

**DOI:** 10.18632/oncotarget.28476

**Published:** 2023-07-20

**Authors:** Takahiro Yamakawa, Guoxiang Zhang, Liza Bengrine Najjar, Chun Li, Keiichi Itakura

**Affiliations:** ^1^Center for RNA Biology and Therapeutics, Beckman Research Institute of City of Hope, Duarte, CA 91010, USA

**Keywords:** KIAA0930, cancer cachexia, muscle atrophy, inflammatory cytokines/chemokines, protein secretion

## Abstract

Patients with cancer cachexia have a poor prognosis and impaired quality of life. Numerous studies using preclinical models have shown that inflammatory cytokines play an important role in the development of cancer cachexia; however, no clinical trial targeting cytokines has been successful. Therefore, it is essential to identify molecular mechanisms to develop anti-cachexia therapies. Here we identified the uncharacterized transcript *KIAA0930* as a candidate cachexic factor based on analyses of microarray datasets and an *in vitro* muscle atrophy assay. While conditioned media from pancreatic, colorectal, gastric, and tongue cancer cells caused muscle atrophy *in vitro*, conditioned medium from *KIAA0930* knockdown cells did not. The PANC-1 orthotopic xenograft study showed that the tibialis anterior muscle weight and cross-sectional area were increased in mice bearing *KIAA0930* knockdown cells compared to control mice. Interestingly, *KIAA0930* knockdown did not cause consistent changes in the secretion of inflammatory cytokines/chemokines from a variety of cancer cell lines. An initial characterization experiment showed that KIAA0930 is localized in the cytosol and not secreted from cells. These data suggest that the action of KIAA0930 is independent of the expression of cytokines/chemokines and that KIAA0930 could be a novel therapeutic target for cachexia.

## INTRODUCTION

Cancer treatment has been dramatically improved for the past few decades. While only a few options, such as surgery, radiation therapy, and chemotherapy were available in the past, extensive basic research and clinical trials lead to a development of a variety of new therapeutic strategies, including targeting cancer therapies [1]. Many types of the targeting drugs such as monoclonal antibodies, immunotherapy, and small compounds have been shown to be more effective than previous therapies [2, 3]. For example, the first-in-class KRAS G12C inhibitor (sotorasib) showed to be more successful for lung cancer treatment than docetaxel [4], and inhibition of the programmed cell death receptor (PD-1) or its ligand 1 (PD-L1) by antibodies was effective for treatment of multiple types of cancers [5]. However, it is still challenging to invent effective therapies for metastatic, drug-resistant and recurrent cancers, and cancer-caused complications such as cancer cachexia (CC). To achieve this difficult goal, an innovative approach to reveal a new target is highly desirable.

We took a unique strategy to explore a new target from uncharacterized transcripts. Using publicly available microarray datasets and literature databases, candidate transcripts that were upregulated in cancer patients and had not been investigated in the past were selected. The selected transcripts were screened for several cancer phenotype using the pancreatic cancer cell line PANC-1 with the transcripts being knocked down. We found that the expression of the uncharacterized transcript *KIAA0930* (*C22orf9*) is significantly higher in pancreatic, colorectal, gastric, and tongue cancer tissues than in normal tissues in clinical microarray datasets and that *KIAA0930* knockdown reduces muscle atrophy *in vitro* and *in vivo*. Interestingly, the effect cannot be explained by changes in the expression of inflammatory cytokines/chemokines, which have historically been thought to be major CC-inducing factors in preclinical models [6–14] and human cancers [15]. These features of KIAA0930 could lead to unique cachexia therapeutics. We here report the initial characterization of KIAA0930.

## RESULTS

### Selection of the uncharacterized transcript, *KIAA0930*

Although almost all transcripts have been cloned and sequenced, many transcripts remain uncharacterized, and those encoding proteins with unknown functions are named with the convention “*chromosome open reading frame*”, for example, *chromosome 5 open reading frame 13* (*C5orf13*). We speculated that these uncharacterized transcripts could play a role in cancer progress and that characterization of the transcripts may lead to development of new cancer therapeutic agents. We first identified these transcripts, whose functions have not been investigated, from a publicly available microarray dataset of pancreatic cancer (GSE16515). The transcripts that are significantly upregulated (more than 1.5-fold) in cancer patients compared to normal groups were listed, and top 5 highly expressed transcripts *C5orf13*, *C7orf42*, *C9orf16*, *C15orf48*, and *C22orf9* were selected for further experiments. To test whether these transcripts regulate any cancer phenotype *in vitro*, they were transiently knocked down in PANC-1 cells, and cell proliferation, migration, and muscle atrophy assays were carried out *in vitro*. No significant effects were found in cell proliferation and migration when all transcripts were suppressed (data not shown). For the next characterization of these transcripts, the *in vitro* muscle atrophy assay was carried out under conditions as described for PANC-1 cells (Supplementary Figure 1A and 1B). Interestingly, we found that conditioned medium (CM) from *C22orf9* (*KIAA0930*)-knockdown PANC-1 cells suppressed C2C12 myotube atrophy but CM from cells with knockdown of the other transcripts did not (Supplementary Table 1).

### *KIAA0930* expression is upregulated in multiple primary cancers, and conditioned medium from *KIAA0930* knockdown cell lines consistently reverses C2C12 myotube atrophy *in vitro*

**Figure 1:**
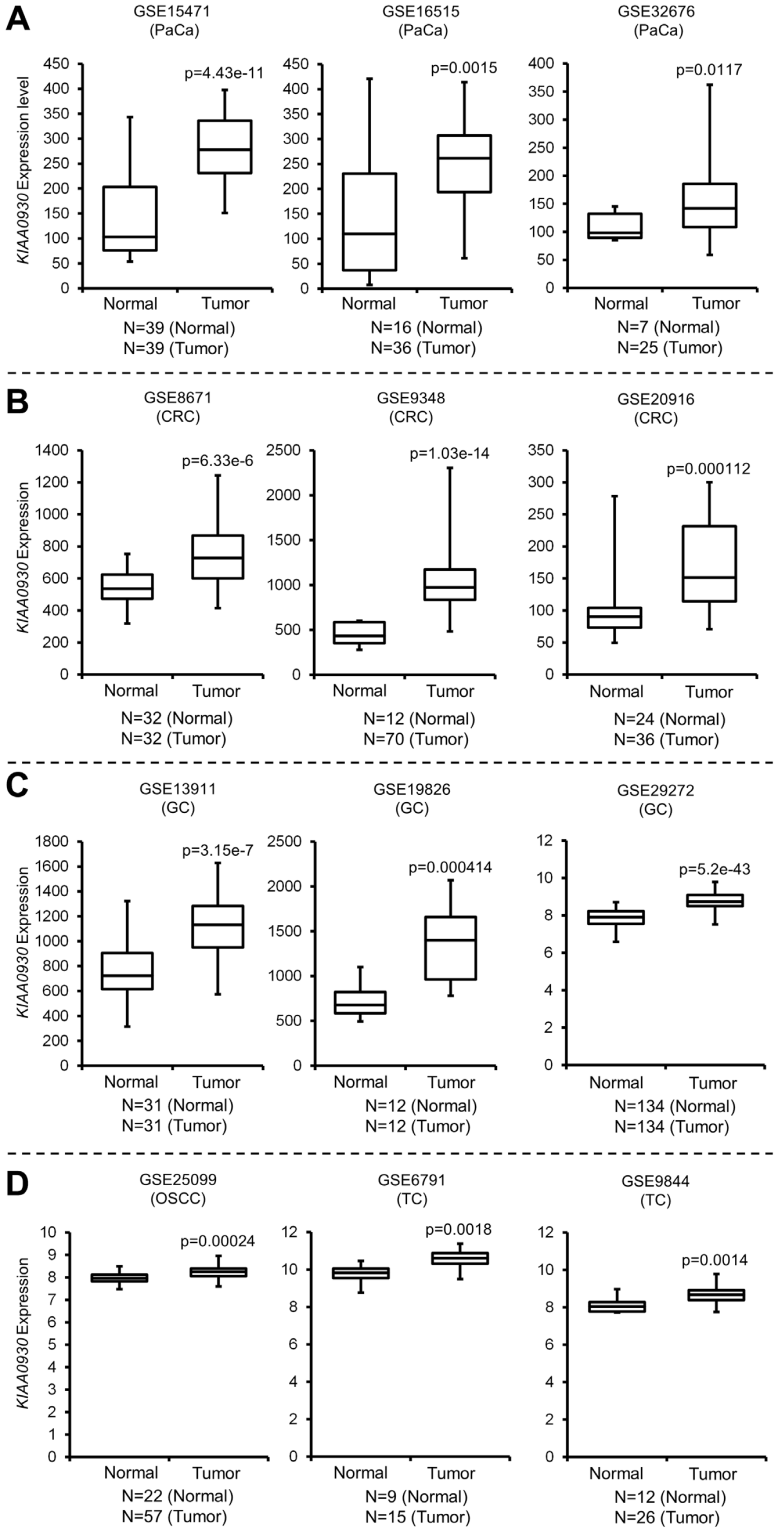
*KIAA0930* is upregulated in pancreatic, colorectal, gastric and tongue cancers. Box and whisker dot plots showing that *KIAA0930* expression is increased in (**A**) pancreatic (PaCa), (**B**) colorectal (CRC), (**C**) gastric (GC), (**D**) oral squamous cell carcinoma (OSCC) and tongue (TC) cancers in multiple publicly available microarray datasets.

**Figure 2:**
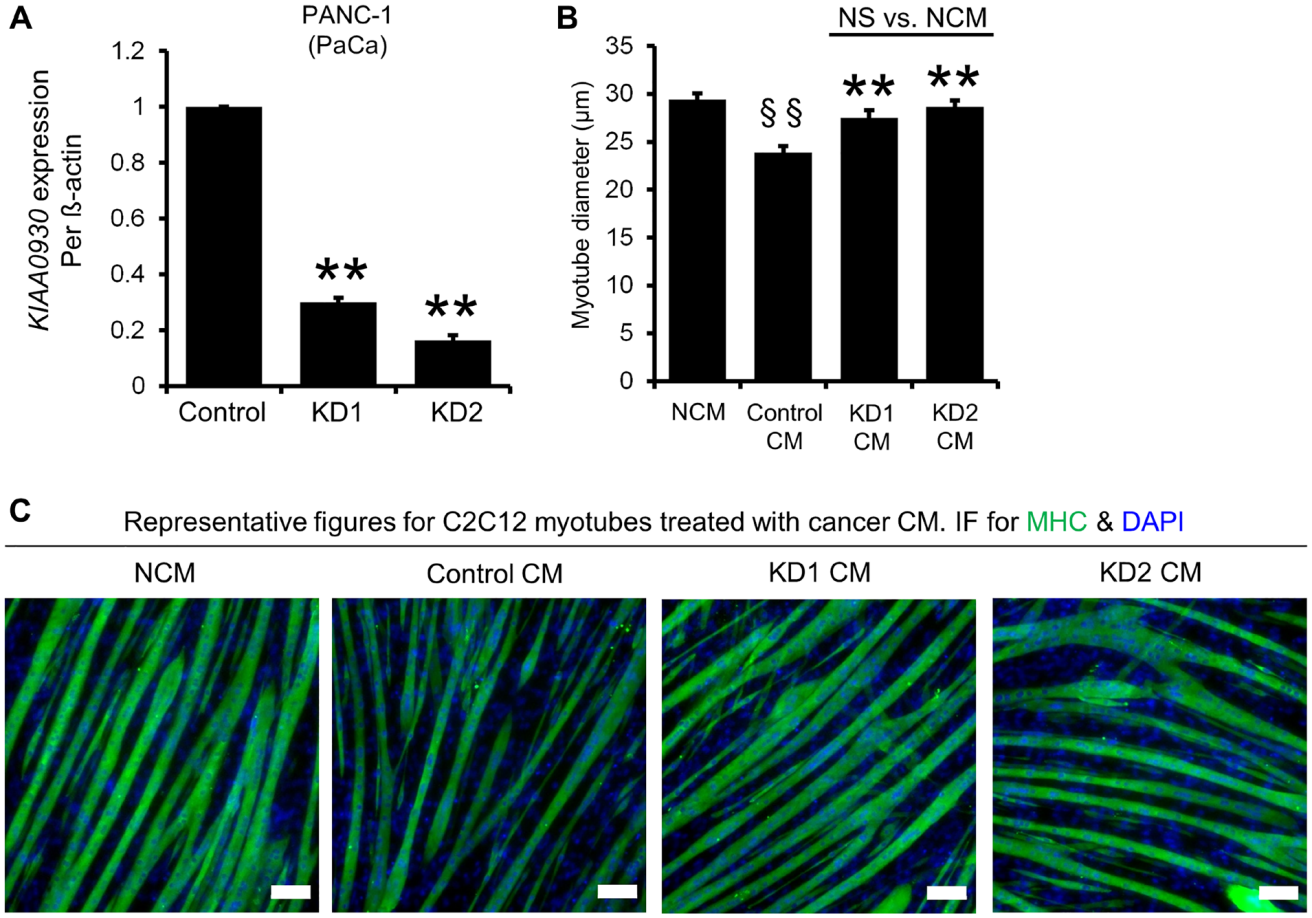
ShRNAs targeting *KIAA0930* decrease *KIAA0930* mRNA expression and suppress muscle atrophy in PANC-1 cells *in vitro*. PANC-1 cells were transduced with lentiviral vectors expressing shRNAs targeting *KIAA0930* (KD1 and KD2) or a nontargeted shRNA (Control) and selected with blasticidin to establish cells stably expressing these shRNAs. (**A**) Total RNA was extracted and subjected to real-time RT‒PCR. *KIAA0930* mRNA expression was normalized to ß-actin expression. (**B**) Control, KD1, and KD2 PANC-1 cells were seeded in 12-well plates, and conditioned medium (CM) and nonconditioned medium (NCM) were collected. Differentiated C2C12 myotubes were cultured with NCM and CM at a concentration of 10% for 2 days, fixed, and stained for MHC and nuclei (DAPI). The diameter of MHC-positive myotubes was measured. (**C**) Representative images of MHC and DAPI staining are also shown (scale bar = 100 μm). The mRNA expression data are shown as the mean ± S.E. from 3–11 independent experiments. ^**^ *p* < 0.01 vs. control. Myotube diameter data shown are representative of at least three independent experiments (mean ± S.E.; *n* = 39–46 myotubes). ^**^ *p* < 0.01 vs. Control CM; ^§§^ *p* < 0.01 vs. NCM. Abbreviation: NS: not significant.

Since the aforementioned data suggested that knockdown of *KIAA0930* in PANC-1 cells inhibited myotube atrophy, we further analyzed the expression of *KIAA0930* in microarray datasets from multiple cancer types to examine whether *KIAA0930* is a common factor among different cancers. *KIAA0930* expression was upregulated in pancreatic cancer (PaCa; GSE15471, GSE16515, and GSE32676), colorectal cancer (CRC; GSE8671, GSE9348, and GSE20916), gastric cancer (GC; GSE13911, GSE19826, and GSE29272), oral squamous cell carcinoma (OSCC; GSE25099) and tongue cancer (TC; GSE6791 and GSE9844) (Figure 1A–1D), which have a high risk for CC [16, 17]. These data suggested that KIAA0930 may confer a cachexic phenotype on cells in a variety of cancers. To examine this hypothesis, we carried out an *in vitro* muscle atrophy assay using CM from several cell lines derived from multiple cancer types (Figure 2B, Supplementary Figure 1C and 1D). Compared to NCM, the myotube diameters were decreased by treatment with CM from all control cancer cells listed in Figure S1C in addition to PANC-1 cells, indicating that these cancer cells secrete atrophy-inducing factors (compare the left two columns). In contrast, CM from the kidney cancer cell lines 786-O and Caki-1 did not cause muscle atrophy under the same conditions (Supplementary Figure 1D). Next, to investigate the function of KIAA0930 in these cancer cell lines, we established two stable *KIAA0930* knockdown cell lines using lentiviral vectors encoding an shRNA targeting *KIAA0930*. Sufficient knockdown of *KIAA0930* in comparison to the nontargeted control cell lines was confirmed for all shRNAs and cell lines except for KD2 in CAL 27 cells (Figure 2A and Supplementary Figure 2A). Remarkably, CM from *KIAA0930* knockdown cells did not reduce the myotube diameter; the myotube diameters were the same as those in the corresponding NCM-treated cells (Figure 2B and 2C for PANC-1 cells, and Supplementary Figure 1C for other cells; compare the second and third/fourth columns). Importantly, the effect of KIAA0930 knockdown was consistently observed in all cancer cells tested except for KD2 in CAL 27 cells. Notably, as described in Supplementary Figure 2A, CAL 27 KD2 cells did not show a significant reduction in the expression of *KIAA0930* compared to the corresponding control cells.

CC has also been reported to suppress myotube differentiation (myogenesis) [18, 19]. We therefore examined the effect of CM from KIAA0930 knockdown cells on myogenesis in C2C12 cells and found that CM from *KIAA0930* knockdown cells did not suppress myogenesis (Supplementary Figure 2B). Similar to the observations regarding the myotube diameter, myogenesis did not appear to be affected in CAL 27 KD2 cells. Collectively, these data strongly suggest that a reduction in *KIAA0930* expression in cancer cells suppresses muscle atrophy and that KIAA0930 plays an essential role in myotube atrophy *in vitro*.

We also investigated whether knockdown of *KIAA0930* affects cell proliferation and migration. *KIAA0930* knockdown (with KD1 and KD2) increased the proliferation of Capan-2 cells but decreased the proliferation of Panc 02.13 and MKN45 cells in monolayer culture (2D) (Supplementary Figure 3A). Knockdown of *KIAA0930* did not change the proliferation of Mia PaCa-2, HT29, and CAL 27 KD1 cells, and *KIAA0930* KD1 and KD2 differentially affected the proliferation of PANC-1, CFPAC-1, and HCT116 cells (Supplementary Figure 3A). Knockdown of *KIAA0930* also resulted in variable and inconsistent effects on anchorage-independent (3D) cell proliferation and cell migration (Supplementary Figure 3B and 3C respectively). Overall, KIAA0930’s function in muscle atrophy is consistently observed, but not cell proliferation and migration *in vitro*. We therefore further investigated the function of muscle atrophy *in vivo*.

### Knockdown of *KIAA0930* alleviates muscle atrophy in a PANC-1 orthotopic xenograft model.

**Figure 3:**
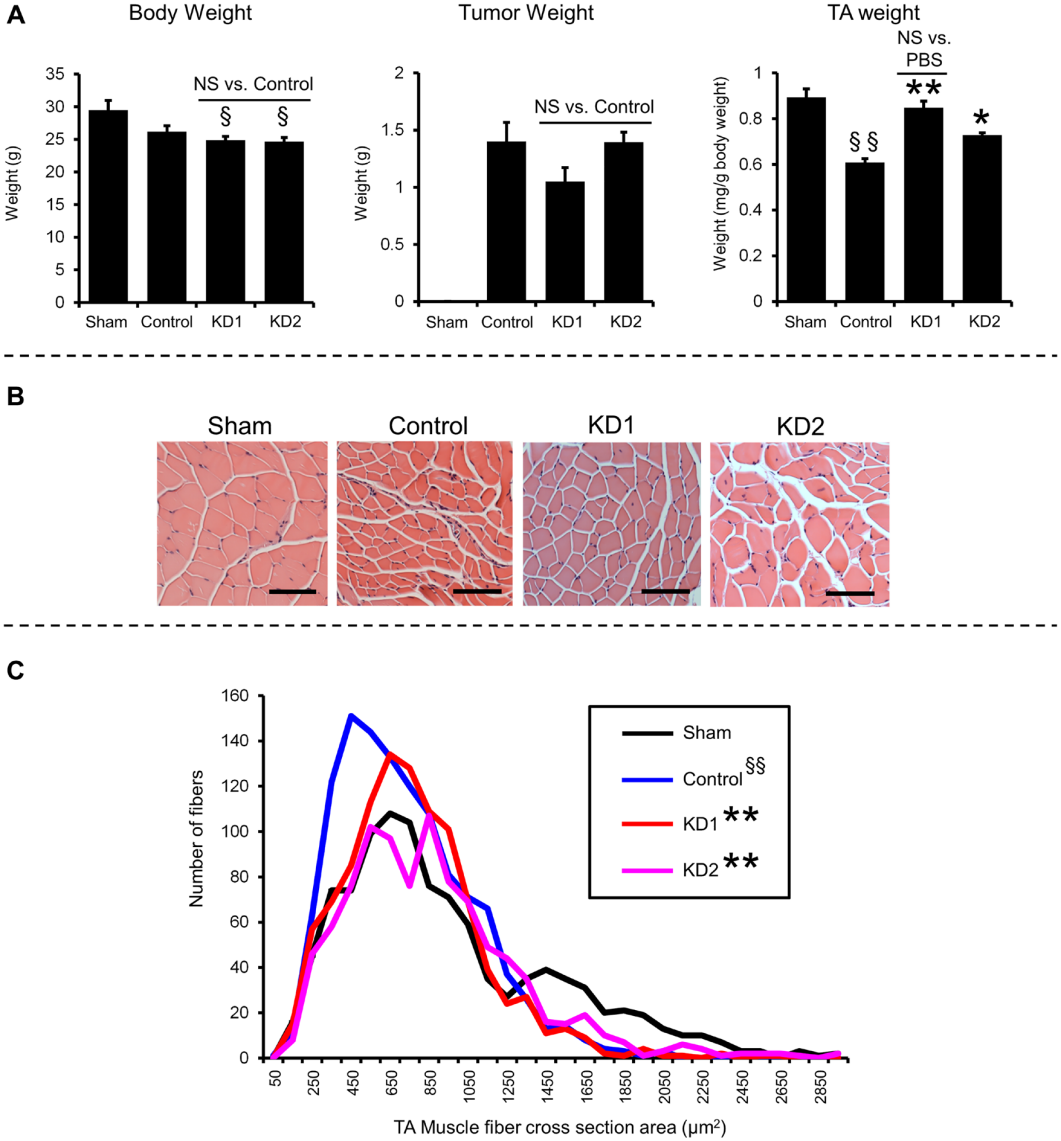
*KIAA0930* knockdown alleviates muscle atrophy in a PANC-1 orthotopic xenograft model. PANC-1 control, KD1, and KD2 cells were inoculated into the pancreas of NSG mice, and tumor-bearing mice were maintained for up to 8 weeks. (**A**) Body, tumor, and TA muscle weights. (**B**) Representative images of TA muscles with H-E staining (scale bar = 100 μm). (**C**) Frequency distribution of the TA muscle fiber cross-sectional area. The data are shown as the mean ± S.E. values (*n* = 5–6 mice). ^**^ *p* < 0.01; ^*^ *p* < 0.05 vs. Control group, ^§§^ *p* < 0.01; ^§^ *p* < 0.05 vs. Sham-operated group. Abbreviation: NS: not significant.

To show that the suppression of myotube atrophy *in vitro* by knockdown of KIAA0930 could be reproduced *in vivo*, we conducted a xenograft assay using orthotopic inoculation of cancer cells following the published method [20]. Nontargeted control, KD1, or KD2 PANC-1 cells were injected into the pancreas of NSG mice, and these tumor-bearing and sham-operated mice were maintained for 8 weeks. Body, tumor, and tibialis anterior (TA) muscle weights and the cross-sectional area of the TA muscle were measured. Control PANC-1 cell inoculation reduced the TA muscle weight compared to those in the sham-operated group (Figure 3A (right); compare the left two columns). Tumor weights in mice inoculated with PANC-1 control cells were not different from those in mice inoculated with KD1 and KD2 cells, indicating that *KIAA0930* knockdown did not affect tumor growth in the xenograft model (Figure 3A (middle)). Although the body weights of mice bearing PANC-1 KD1 and KD2 cells were not increased compared to those of mice bearing control cells, the TA muscle weights in the KD1 and KD2 groups were significantly higher than that in the control group (Figure 3A (right)). We also examined TA muscle myofibers (Figure 3B) and found that the fiber cross-sectional areas in the KD1 and KD2 groups were significantly increased compared to that in the control group (Figure 3C). Thus, our data indicated that orthotopic inoculation of *KIAA0930* knockdown PANC-1 cells reduces muscle atrophy in mice.

Since it has been shown that inflammatory cytokines/chemokines are key factors that cause muscle atrophy in preclinical models [6, 9, 13, 14, 21, 22], we next examined human and mouse cytokine/chemokine levels in plasma. We did not observe any significant differences in the expression levels of human granulocyte-macrophage colony stimulating factor (GM-CSF), interleukin-8 (IL-8), IL-15, growth-regulated oncogene (GRO), FGF2, and monocyte chemoattractant protein-1 (MCP-1, also known as CCL2) between control and knockdown groups (Supplementary Figure 4A). Mouse Il-6 and Ccl2 levels in the plasma from the control PANC-1 cell-inoculated mice were higher than those in the sham-operated group (Supplementary Figure 4B). However, the cytokine/chemokine levels in both the KD1 and KD2 PANC-1 cell-inoculated groups were not significantly different from those in the control group (Supplementary Figure 4B). These data suggest that knockdown of *KIAA0930* does not affect the levels of cytokines/chemokines secreted from PANC-1 cells or secondarily induced in mouse organs. We further investigated gene expression in TA muscles from these mice. In line with a previous report [20], the mRNA expression of the atrophy-related genes *Atrogin-1*, *muscle RING-finger protein-1* (*Murf1*), and *Forkhead box O1* and *O3* (*Foxo1* and *Foxo3a*) was induced in the control PANC-1 group compared to the sham-operated group due to the increased expression of cytokines/chemokines (Supplementary Figure 4C; compare the left two columns). However, knockdown of *KIAA0930* in PANC-1 cells did not result in any changes in the expression of these atrophy-related genes in comparison to those in control PANC-1 cells (Supplementary Figure 4C). Collectively, these data suggest that the anti-atrophic effect of *KIAA0930* knockdown may be unrelated to changes in cytokine/chemokine expression.

### Cytokine/chemokine contents in CM do not account for the effect on myotube atrophy

**Figure 4:**
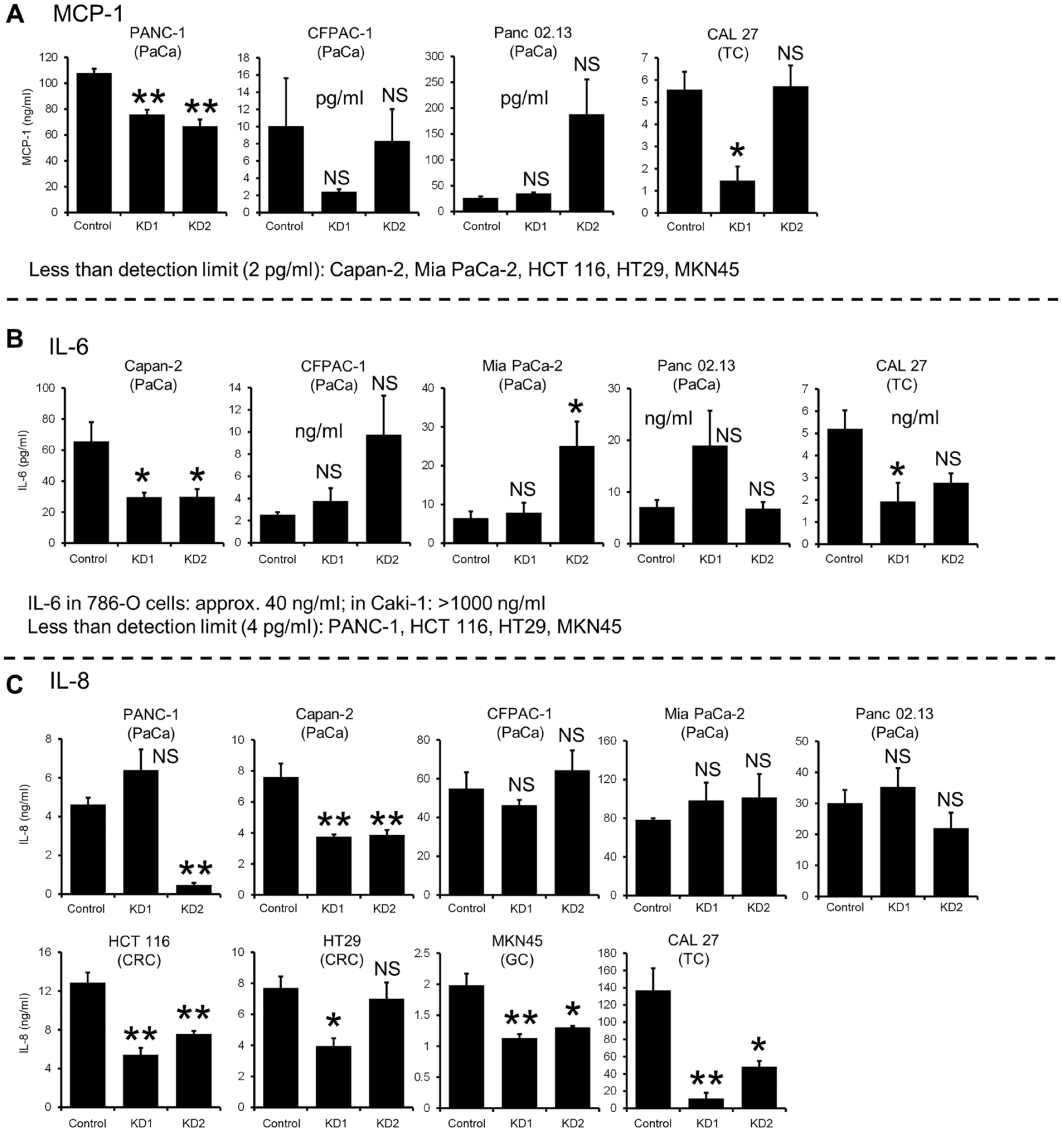
Cytokine/chemokine secretion from KIAA0930 knockdown cancer cells. The conditioned media from control and KIAA0930 KD1 and KD2 cells were collected, and cytokine/chemokine contents were measured using ELISA kits. (**A**) MCP-1, less than detection limit (2 pg/ml) in conditioned medium (CM) from Capan-2, Mia PaCa-2, HCT 116, HT29, and MKN45 cells; (**B**) IL-6, approximately 40 and more than 1000 ng/ml in CM from 786-O and Caki-1 cells, respectively, less than detection limit (4 pg/ml) in CM from PANC-1, HCT 116, HT29, and MKN45 cells; (**C**) IL-8; expression levels are shown. The IL-1ß and tumor necrosis factor-alpha (TNFα) expression levels were lower than the detection limits (2 pg/ml and 25 pg/ml, respectively) in CM from all cell lines we used. The data are shown as the mean ± S.E. from three independent experiments. ^*^ *p* < 0.05; ^**^ *p* < 0.01 vs. control. Abbreviation: NS: not significant.

Although muscle atrophy was significantly ameliorated in mice implanted with *KIAA0930* knockdown PANC-1 cells (Figure 3), the human and mouse cytokine/chemokine levels in the plasma were not changed (Supplementary Figure 4A and 4B). To investigate whether knockdown of *KIAA0930* affects cytokine/chemokine secretion *in vitro*, we first analyzed cytokine/chemokine secretion from various cancer cell lines using ELISAs and found that the cytokine/chemokine secretion profiles were completely different among the cell lines (Figure 4A–4C and Supplementary Figure 5A–5C; compare the left column in each Figure). For example, MCP-1 was secreted abundantly from PANC-1 cells and IL-6 from Panc 02.13, 786-O, and Caki-1 cells but not from the other cell lines. In addition, the level of IL-8 secretion was categorized as 1) high secretion (CFPAC-1, Mia PaCa-2, and CAL 27 cells), 2) moderate secretion (Panc 02.13 and HCT 116 cells), and 3) low secretion (PANC-1, Capan-2, HT29, and MKN45 cells). Next, we examined the effect of *KIAA0930* knockdown on the secretion of cytokines/chemokines. As shown in Figure 4A–4C and Supplementary Figure 5A–5C, the effect was different between KD1 and KD2 and among the cell lines (compare the left two columns and right two columns). Our observation of consistent and significant inhibition of muscle atrophy in C2C12 myotubes treated with CM from a variety of *KIAA0930* knockdown cells (Figure 2B and Supplementary Figure 1C) strongly suggests that the effect of *KIAA0930* knockdown on the inhibition of muscle atrophy is independent of the changes in cytokine/chemokine secretion.

### Initial characterization of the KIAA0930 protein

**Figure 5:**
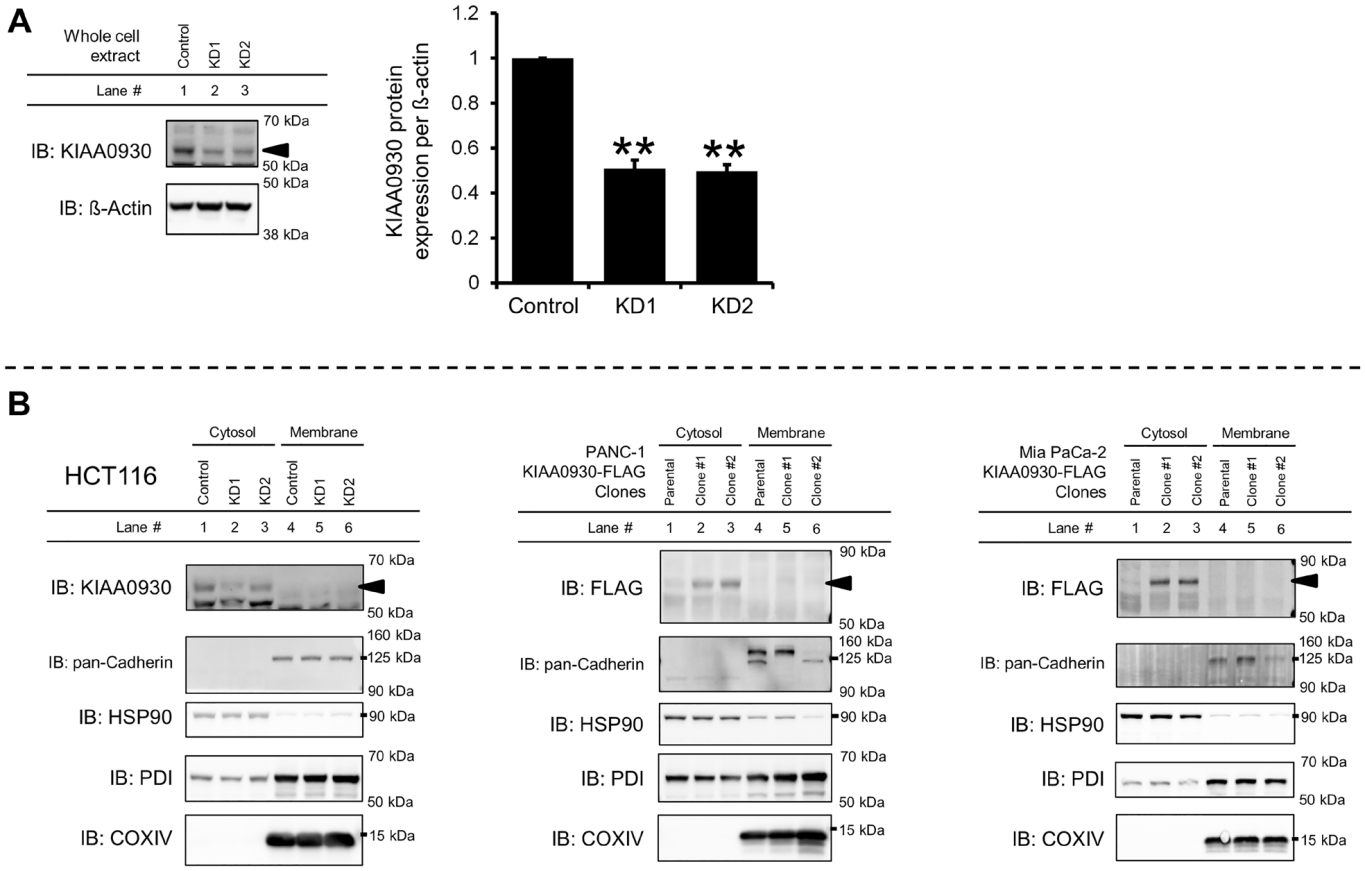
KIAA0930 is a cytosolic protein. (**A**) Detection of endogenous KIAA0930 protein in HCT116 cells. *Left*: representative images of Western blotting. *Right*: protein expression of KIAA0930 normalized to ß-actin in HCT116 cells. The data are shown as the mean ± S.E. from three independent experiments. ^**^ *p* < 0.01 vs. control. (**B**) Subcellular localization of KIAA0930 in HCT116 cells and in PANC-1 and Mia PaCa-2 KIAA0930-FLAG clones. Cellular proteins were separated into the cytosolic and membrane fractions and subjected to SDS‒PAGE. The following proteins were examined to show appropriate fractionation. Abbreviations: HSP90: cytosol; pan-Cadherin: plasma membrane; PDI: endoplasmic reticulum membrane; COXIV: mitochondrial membrane.

**Figure 6:**
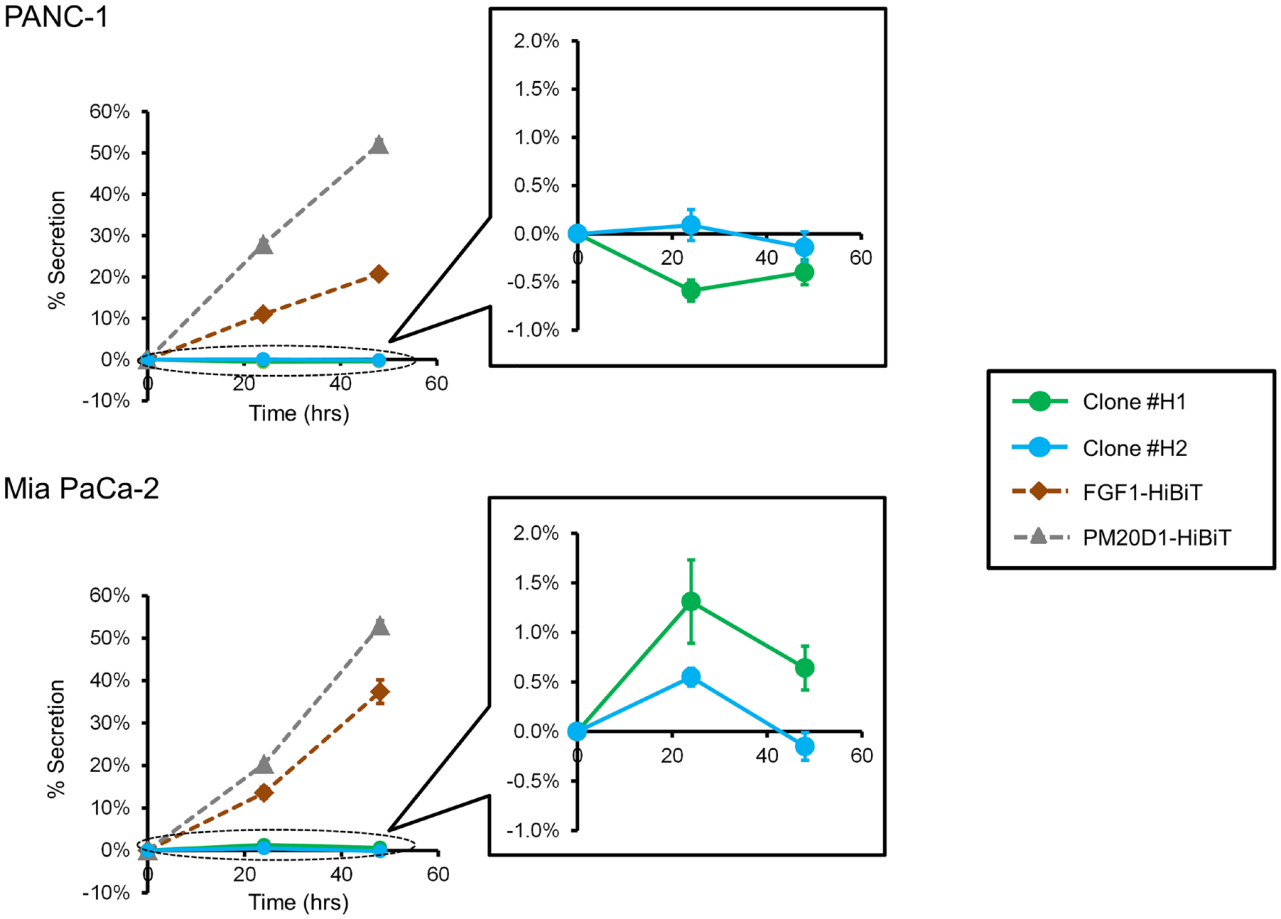
KIAA0930 is a nonsecreted protein. Supernatants and lysates were collected from PANC-1 and Mia PaCa-2 HiBiT clones, and luminescence signals from the HiBiT tag were measured according to the Materials and Methods section. The cells were also transfected with the FGF1-HiBiT or PM20D1-HiBiT plasmid, and the signals were measured as described above. The percent secretion per well was calculated with the following formula: 100 × (signal in supernatant) ÷ (signal in supernatant + Cell lysate). The data shown are representative of three independent experiments (mean ± S.E.; *n* = 6).

To initially characterize the KIAA0930 protein, we conducted the following investigations. AlphaFold, which enables highly accurate prediction of protein structures [23], was used to generate a three-dimensional structure of KIAA0930 (Supplementary Figure 6A), which does not contain any known domains. Since protein analysis tools suggested that KIAA0930 contains neither a signal peptide nor a transmembrane domain [24, 25], we first examined the subcellular localization of the KIAA0930 protein. In HCT 116 cells, the KIAA0930 protein was successfully detected using Western blotting with a minimum number of nonspecific bands (Figure 5A); however, we were unable to specify the band corresponding to the KIAA0930 protein due to the lower specificity of the antibody in the other cells (Supplementary Figure 6B for PANC-1 and Mia PaCa-2 cells and data not shown). To overcome the lower specificity, we established PANC-1 and Mia PaCa-2 cell lines with endogenously FLAG-tagged *KIAA0930* using CRISPR/Cas9 gene editing with homology-directed recombination (Supplementary Figure 6C and 6D) [26]. Notably, the band corresponding to FLAG-tagged KIAA0930 appeared at a higher molecular weight (70 kDa) than native KIAA0930 (approximately 55 kDa) because of the addition of the 3XFLAG tag, as described previously [27]. The data showed that the KIAA0930 protein is predominantly localized in the cytosol (Figure 5B). To examine whether the KIAA0930 protein is a secreted protein, endogenous tagging of *KIAA0930* with a luminescent peptide (HiBiT) was carried out in PANC-1 and Mia PaCa-2 cells (Supplementary Figure 7A and 7B) [28]. HiBiT-tagged cells were seeded and cultured for up to 48 hr, and CM and cell lysates were harvested for the detection of HiBiT in the CM or lysates. While HiBiT signals were hardly detected in the CM (less than 2% of the total signal) and did not increase further in a time-dependent manner in HiBiT-tagged clones (Figure 6), the signals were detected in cell lysates from the clones (Supplementary Table 2). In validation of this assay, we identified FGF1 and PM20D1 exclusively in CM from cells transiently transfected with plasmids expressing these HiBiT-tagged secreted proteins (Figure 6). It can also be possible that extracellular vesicles from cancer cells may contain *KIAA0930* transcript, which could be transported into C2C12 myotubes. To examine this, we treated C2C12 myotubes with CM from HiBiT-tagged PANC-1 or Mia PaCa-2 cells, and measured HiBiT signals in myotubes. However, the levels of signals in myotubes treated with CM from HiBiT-tagged cancer cells were the same as those with parental cells (Supplementary Table 3). These data suggest that conditioned media do not contain *KIAA0930* transcript. Collectively, these results show that KIAA0930 is an uncharacterized protein that is not secreted but is localized in the cytosol.

## DISCUSSION

CC is a multifactorial syndrome characterized by changes in body composition in patients with advanced cancer. Although many phenotypes, including loss of appetite and wasting of adipose tissue, are observed in CC, muscle wasting is a representative feature of CC [29]. Over the past more than 20 years, inflammatory cytokines/chemokines have been a primary focus of research to reveal the mechanisms underlying CC. However, the results of clinical studies based on anti-inflammatory cytokine treatment such as anti-TNFα and anti-IL-6 monoclonal antibodies, are disappointing [30, 31], and the mechanism by which cytokines cause CC is not completely clear. Therefore, we used a unique approach to search for a novel target for CC by analyzing publicly available microarray datasets and literature databases. We identified several candidates, and further study demonstrated that knockdown of the uncharacterized transcript *KIAA0930* in pancreatic cancer cells alleviated muscle atrophy *in vitro* and *in vivo*. Importantly, the effect was unrelated to changes in the levels of inflammatory cytokines/chemokines, indicating that KIAA0930 could be a novel target for anti-cachexia therapy.

Since skeletal muscle atrophy is one of the most prominent symptoms of CC [29], we examined myotube atrophy by measuring the myotube diameter *in vitro* for the selection of candidates. The CM from a variety of cancer cells including pancreatic, colorectal, gastric, and tongue cancer cell lines showed atrophy (Figure 2B, 2C, and Supplementary Figure 1C). Importantly, *KIAA0930* knockdown inhibited muscle atrophy in these cells. We also found that 786-O and Caki-1 cells did not exhibit muscle atrophic phenotype (Supplementary Figure 1D), although the expression level of *KIAA0930* in these cells is comparable to the atrophic cancer cells shown in Figure 2 and Supplementary Figure 1C (GSE36133). These data suggest that factor(s) other than KIAA0930 are required for the acquisition of muscle atrophic phenotype in the cells.

Based on preclinical studies regarding CC, it was hypothesized that overexpressed inflammatory cytokines such as IL-1α [11], IL-1ß [11], IL-6 [9], TNFα [10, 11], and TGFß [8, 21, 22] induce skeletal muscle atrophy through increased expression of E3 ubiquitin ligases, Atrogin-1 and Murf1 [32]. Indeed, these two atrophy-related genes were upregulated in TA muscle from PANC-1-inoculated control mice (Supplementary Figure 4C), suggesting that these cytokines contributed to muscle atrophy in our xenograft model. Remarkably, however, we found that there were no significant differences in the expression of these two atrophy-related genes in the muscle between the *KIAA0930* knockdown and control groups (Supplementary Figure 4C), even though atrophy was significantly suppressed in the knockdown group (Figure 3). Consistent with this finding, the cytokine/chemokine concentrations in plasma were not changed by knockdown of *KIAA0930* compared to the control group (Supplementary Figure 4A and 4B). This finding is not surprising; it was reported that the expression levels of atrophy-related genes were not correlated with muscle atrophy in human pancreatic cancer PDX models [33] and human cachexic patients [6, 34]. Although the detection limitation exists, these data suggest that factors other than Atrogin-1 and Murf-1 also could be involved in muscle atrophy and that the suppression of KIAA0930 in cancers may alleviate muscle atrophy without changes in the expression levels of cytokines/chemokines.

It was shown that CM from 786-O and Caki-1 cells did not exhibit muscle atrophy (Supplementary Figure 1D), even though a large amount of IL-6 was secreted from these cells (Figure 4B). IL-6 is known to induce muscle atrophy *in vitro* and preclinical models [9]. In addition, it seems that profile of individual cytokine/chemokine secretion from various cell lines was not associated with muscle atrophy activity *in vitro* (Figure 2B and Supplementary Figure 1C, and Figure 4A–4C and Supplementary Figure 5A–5C). These data show that inflammatory cytokines/chemokines are not only the factors to induce muscle atrophy and that stimulatory and/or inhibitory factors may involve the atrophy.

We assume that cancer cells secrete communication mediators such as proteins to induce skeletal muscle atrophy. Most secreted proteins, including cytokines and secreted enzymes such as PM20D1, possess signal peptides (also called “leader sequences”), which are recognized by the endoplasmic reticulum (ER) and transported to the trans-Golgi complex prior to the formation of a vesicular carrier for release from the plasma membrane, which prevents any modification from occurring in the cytosol (the conventional protein secretion pathway) [35, 36]. Accumulating evidence shows that some secretory proteins, such as FGF1 and FGF2, use alternative pathways that do not involve ER-to-Golgi transport, a route called the unconventional secretory pathway [37–39]. In this pathway, proteins are secreted from the cytosol through pores on the plasma membrane or packaged into membrane-bound organelles, including endosomes and exosomes, and are secreted in extracellular vesicles, which also contain nucleic acids such as DNA and microRNAs, amino acids, and metabolites [40]. Since the KIAA0930 protein is localized in the cytosol (Figure 5B), we speculate that KIAA0930 may regulate a secretion process via the unconventional pathway and might confer a muscle atrophic phenotype in diverse cancers. Further investigation is needed to identify the precise molecular mechanisms by which KIAA0930 controls CC and to investigate the factors that contribute to muscle atrophy. In conclusion, we demonstrate a function of the uncharacterized transcript *KIAA0930* in cachexia in cancers, with a mechanism that could not be linked to the expression levels of inflammatory cytokines/chemokines. We believe that KIAA0930 would be a novel cachexia therapeutic target.

## MATERIALS AND METHODS

### Analysis of microarray datasets

Publicly available pancreatic cancer (GSE15471, GSE16515, and GSE32676), colorectal cancer (GSE8671, GSE9348, and GSE20916), gastric cancer (GSE13911, GSE19826, and GSE29272), tongue cancer (GSE6791 and GSE9844), and oral squamous cell carcinoma (GSE25099) data sets were downloaded from GEO (Gene Expression Omnibus). A two-sample *t* test was carried out using EZR [41], which is a graphical user interface for R (The R Foundation for Statistical Computing, Vienna, Austria).

### Cell line culture and small interfering RNA transfection

The human pancreatic cancer (PaCa) cell lines PANC-1, Capan-2, CFPAC-1, Mia PaCa-2 and Panc 02.13, the human colorectal cancer (CRC) cell lines HCT116 and HT29, the human tongue cancer (TC) cell line CAL 27, the kidney cancer cell lines 786-O and Caki-1, and the mouse myoblast cell line C2C12 were purchased from the American Type Culture Collection (Manassas, VA, USA). The human gastric cancer (GC) cell line MKN45 was purchased from the Japanese Collection of Research Bioresources Cell Bank (Osaka, Japan). The 293T cell line was purchased from Biosettia (San Diego, CA, USA). PANC-1, CAL 27, C2C12, and 293T cells were maintained in DME high-glucose medium (Irvine Scientific; Santa Ana, CA, USA) supplemented with 10% fetal bovine serum (FBS, Cytiva; Marlborough, MA, USA). Capan-2, HCT116, HT29, and Caki-1 cells were maintained in McCoy’s 5A medium (Corning; Corning, NY, USA) supplemented with 10% FBS. CFPAC-1 cells were maintained in Iscove’s modified Dulbecco’s medium (Thermo Fisher Scientific; Pittsburgh, PA, USA) supplemented with 10% FBS. The Mia PaCa-2 cell line was maintained in DME high-glucose medium supplemented with 10% FBS and 2.5% horse serum (HS, Thermo Fisher Scientific). The Panc 02.13 cell line was maintained in RPMI-1640 medium (ATCC) supplemented with 15% FBS and 10 U/ml human insulin (MP Biomedicals; Solon, OH, USA). The MKN45 and 786-O cell lines were maintained in RPMI-1640 medium (Irvine Scientific) supplemented with 10% FBS. For transient knockdown, cells were transfected with ON-TARGET Plus Nontargeting Pool and a targeted SMARTpool (Horizon Discovery; Cambridge, United Kingdom) at 50 nM for 24 hr using Lipofectamine 3000 reagent (Thermo Fisher Scientific) according to the manufacturer’s protocol.

### Lentiviral packaging and transduction

The nontargeted (Control) and KIAA0930 shRNA (shKIAA0930 KD1 and KD2) sequences used in this study are shown in Supplementary Table 4. The palindromic oligos were annealed and inserted into the pLV-hU6-EF1a-GFP-Bsd shRNA vector (Biosettia) according to the manufacturer’s instructions. To produce lentiviral particles, 293T cells were transfected with these lentiviral vectors and packaging plasmids (Biosettia) using Lipofectamine 3000 reagent. The culture supernatants were collected and concentrated using Speedy Lentivirus Purification reagent (Applied Biological Materials, Inc.; Richmond, British Columbia, Canada). The viral titer was determined using 293T cells as described previously [42–44]. Cancer cells were transduced for 24 hr at an MOI of 10 in the presence of 6 μg/mL polybrene and selected with blasticidin.

### Plasmid construction

pSpCas9(BB)-2A-GFP (PX458) was a gift from Feng Zhang (Addgene plasmid # 48138; http://n2t.net/addgene:48138; RRID:Addgene_48138). pFETCh_Donor (EMM0021) was a gift from Eric Mendenhall and Richard M. Myers (Addgene plasmid # 63934; http://n2t.net/addgene:63934; RRID:Addgene_63934). The two different guide RNAs targeting a sequence near the 3′-end stop codons of KIAA0930 shown in Supplementary Table 5 were inserted into BbsI-digested PX458 [45]. The 5′- and 3′- homology arm fragments for KIAA0930 were amplified using PCR with the primers (5′- or 3′-HOM FWD/REV) shown in Supplementary Table 6 and purified. These fragments and the BsaI/BbsI-digested pFETCh_Donor plasmid were ligated using Gibson Assembly Master Mix (New England Biolabs; Ipswich, MA) to construct the donor vector for C-terminal FLAG-tagging of KIAA0930 (FLAG_KIAA0930_Donor) [26]. PCR fragments synthesized using 5′-phophorylated primer sets (HiBiT_Donor FWD/REV; Supplementary Table 6) and FLAG_KIAA0930_Donor as a template were self-ligated to construct the donor vector for HiBiT-tagged KIAA0930 (HiBiT_KIAA0930_Donor) [28]. The open reading frames (ORFs) of FGF1 and PM20D1 were amplified using PCR with the primers (ORF_pBiT3.1C FWD/REV) shown in Supplementary Table 6 and Myc-DDK-tagged ORF clones (TrueORF^®^; OriGene Technologies, Inc.; Rockville, MD, USA) as templates. These PCR fragments containing the ORFs were ligated into the HindIII/XhoI-digested pBiT3.1-C [CMV/HiBiT/Blast] vector (Promega; Madison, WI, USA) using Gibson Assembly Master Mix to construct the C-terminal HiBiT-tagged expression plasmids (pBiT3.1-C-FGF1 and PM20D1).

### Establishment of endogenously FLAG- or HiBiT-tagged KIAA0930 clones

PANC-1 and Mia PaCa-2 cells were transfected with either the FLAG_KIAA0930_Donor or HiBiT_KIAA0930_Donor plasmid and PX458-guideRNA#1 or #2 using Lipofectamine 3000 reagent. The cells were then cultured with G-418 until resistant colonies appeared. Single cell-derived colonies were isolated, expanded and stored. To confirm successful tagging in the cells, whole-cell extracts from the clones were subjected to SDS-polyacrylamide gel electrophoresis (SDS‒PAGE), followed by Western blotting for the FLAG or HiBiT tag.

### RNA analysis

Total RNA from cell lines was extracted using Direct-zol RNA Miniprep (Zymo Research; Irvine, CA, USA). Muscle tissues were homogenized with a POLYTRON PT 2500 E homogenizer (Kinematica; Bohemia, NY, USA) in TRIzol reagent (Thermo Fisher Scientific), and total RNA was prepared using the RNeasy Plus Mini Kit (QIAGEN; Germantown, MD, USA). cDNA was synthesized using the PrimeScript RT reagent kit (TaKaRa Bio Inc.; San Jose, CA, USA). Messenger RNA expression levels were determined using real-time RT‒PCR. Real-time RT‒PCR was performed with a CFX96 real-time PCR Detection System using iQ SYBR Green Supermix Reagent (Bio-Rad; Hercules, CA, USA). Messenger RNA expression was normalized to β-actin expression. Primer sets are listed in Supplementary Table 7.

### Protein extraction and immunoprecipitation

Cell monolayers were washed with ice-cold phosphate buffered saline (PBS), and cells were harvested in ice-cold lysis buffer (Cell Signaling Technology; Danvers, MA, USA) supplemented with 1 mM phenylmethylsulfonyl fluoride (PMSF). The lysates were centrifuged at 20,000 × g for 5 min at 4°C. The supernatants were collected as whole-cell extracts and stored at −80°C. Cytosolic and membrane proteins were extracted using a Mem-PER^™^ Plus Membrane Protein Extraction Kit (Thermo Fisher Scientific), and cytosolic proteins were concentrated with Nanosep^®^ Centrifugal Devices with Omega^™^ Membrane 3K (PALL Corp.; Westborough, MA, USA). Protein concentrations were determined using a bicinchoninic acid assay (Thermo Fisher Scientific). For immunoprecipitation, whole-cell extracts (500 μg protein in 500 μL buffer) were incubated with 5 μg of an anti-FLAG antibody (F1804; Millipore Sigma; Burlington, MA, USA) overnight at 4°C with gentle shaking. Thirty microliters of Protein G Magnetic Beads (#9006; Cell Signaling Technology) were then added, and the mixture was incubated for an additional 2 hours at 4°C with gentle shaking. The beads were washed three times with lysis buffer, and the bound proteins were eluted with 20 μL of 150 μg/mL 3X FLAG peptide (ApexBio Technology; Houston, TX, USA) in Tris-buffered saline for 30 min at room temperature with shaking. The eluates were collected and subjected to SDS‒PAGE.

### Western blotting

Samples containing equivalent amounts of protein were electrophoresed on SDS‒PAGE gel. The proteins were then transferred to Immobilon-FL polyvinylidene difluoride membranes (Millipore Sigma) and blocked with Odyssey blocking buffer (LI-COR; Lincoln, NE). The membranes were incubated with primary antibodies in Odyssey blocking buffer supplemented with 0.2% Tween 20. After incubation with fluorescent dye-conjugated secondary antibodies, the proteins of interest were detected using a ChemiDoc MP imaging system (Bio-Rad). The following primary and secondary antibodies were used for Western blot analysis: anti-KIAA0930 (NBP2-84553) from Novus Biologicals (Centennial, CO, USA); anti-FLAG (F1804); anti-HSP90 (#4877) and anti-COXIV (#4850) from Cell Signaling Technology; anti-pan-Cadherin (#71-7100) and anti-ß-actin from Thermo Scientific; anti-PDI (ab2792) and Alexa Fluor 647-conjugated anti-rabbit or anti-mouse IgG from Abcam (Boston, MA, USA). For HiBiT tag detection, proteins were transferred to nitrocellulose membranes (Bio-Rad), and the Nano-Glo^®^ HiBiT Blotting System (Promega) was used.

### Cell proliferation assay *in vitro*

For measurement of cell proliferation in monolayer culture (2D), cells were seeded in a white/clear 96-well plate (Thermo Fisher Scientific) at 1 × 10^3^ cells/well and cultured for 1 day. The cells were then cultured for 6 days, with a medium change on Day 3. The cell number was determined using the CellTiter-Glo^®^ 3D Cell Viability Assay Kit (Promega). For measurement of anchorage-independent cell proliferation (3D culture), cells were seeded in an ultralow attachment 96-well plate (Corning) and cultured for 7 days, and the cell number was determined as described above.

### *In vitro* cell migration assay

An ibidi culture insert (ibidi GmbH, Germany) with two chambers separated by a 500 μm wall was used for measurement of cell migration. The insert was placed in a 24-well plate, and cells were seeded in the two chambers. When the cells were confluent, the insert was removed, and the cells were washed three times with serum-free growth medium. Normal growth medium or serum-free medium was added to the wells, and the cells were cultured for 4–24 hours, depending on the cell line. The cells were fixed with 3.7% formaldehyde in PBS, and the area containing migrated cells was measured.

### Preparation of conditioned medium from cancer cells

Cells were seeded in a 12-well plate at 2 × 10^5^ cells/well (PaCa, GC, and TC cell lines) or 4 × 10^5^ cells/well (CRC cell lines). One day after seeding, the cells were washed and cultured in growth medium (0.6 ml/well). After 3 days, cell culture supernatants were collected, centrifuged at 300 × g for 5 min, and stored at −80°C as conditioned medium (CM). To obtain CM from Panc 02.13 cells, cells were washed and cultured in insulin-free growth medium. Growth medium was also incubated without cells as nonconditioned medium (NCM).

### Measurement of C2C12 myotube atrophy *in vitro*

C2C12 myoblasts were seeded in a 24-well plate (6.25 × 10^4^ cells/0.5 ml/well) and cultured for 2 days. The medium was then switched to DME high-glucose medium supplemented with 2% HS (differentiation medium; DM) and replenished after 1, 3, and 5 days. Five days after DM treatment, NCM or CM from cancer cells were added at a final concentration of 10% in 0.25 ml/well DM. Two days after treatment, cells were fixed with 3.7% formaldehyde in PBS for 15 min and blocked with 5% goat serum and 0.3% Triton X-100 in PBS for 1 hour at room temperature. Then, the monolayer was incubated first with an anti-myosin heavy chain antibody (anti-MHC, from R&D Systems; Minneapolis, MN, USA) overnight at 4°C and then with Alexa Fluor 647 (AF647)-conjugated goat anti-mouse IgG (Abcam) and DAPI (Thermo Fisher Scientific) for nuclear staining. MHC/AF647-stained myotubes were photographed using a Zeiss Observer Z1 (ZEISS; Pleasanton, CA) at ×10. The diameters of a total of 40–64 myotubes in 4 random fields were measured using ZEN Imaging Software (ZEISS).

### Measurement of C2C12 myogenesis inhibition *in vitro*

C2C12 myoblasts were seeded and cultured as described above. The medium was changed to DM containing NCM or CM from cancer cells at a final concentration of 2% for Panc 02.13 and CFPAC-1 cells and 10% for other cells on the day of differentiation initiation (designated Day 0), Day 1, and Day 3. On Day 5, cells were fixed, stained for MHC, and analyzed as described above.

### Xenograft assay

Male NOD-SCID IL2Rgamma^null^ (NSG) mice at approximately 8 weeks of age were obtained from The Jackson Laboratory. The mouse experiments were approved by the Institutional Animal Care and Use Committee (IACUC) at the City of Hope. PANC-1 cells (5 × 10^6^) were suspended in 100 μL of serum-free DME high-glucose medium containing 50% Matrigel and implanted orthotopically into the pancreas [20]. PBS was also injected into the pancreas as a sham operation. The mice were maintained for up to 60 days after inoculation, with body weight measured at least once a week. At the time of euthanization, plasma, skeletal muscle, and pancreata with tumors were harvested, weighed, snap frozen in liquid nitrogen, and stored at −80 °C. The tibialis anterior (TA) muscle was fixed with 10% formalin and embedded in paraffin, and cross-sections of the TA muscle were stained with hematoxylin and eosin (H-E). Images were acquired using a Zeiss Observer II (ZEISS). Cross-sectional areas were measured using Image-Pro Premier (Media Cybernetics; Rockville, Maryland).

### Measurement of cytokines/chemokines in cell culture supernatants and mouse plasma

Cytokine/chemokine contents in culture supernatants were measured using sandwich ELISA kits (Ray Biotech; Peachtree Corners, GA, USA) according to the manufacturer’s protocol. Cytokines/chemokines in mouse plasma were measured using a MILLIPLEX^®^ Human Cytokine/Chemokine Magnetic Bead Panel – Premixed 41 Plex – Immunology Multiplex Assay (HCYTMAG60PMX41BK; Millipore Sigma) and a MILLIPLEX^®^ Mouse Cytokine/Chemokine Magnetic Bead Panel – Premixed 32 Plex – Immunology Multiplex Assay (MCYTMAG-70K-PX32; Millipore Sigma).

### Measurement of KIAA0930 protein secretion

Parental PANC-1 and Mia PaCa-2 cells and their clones expressing endogenously HiBiT-tagged KIAA0930 were seeded in a 24-well plate (1 × 10^5^ cells/0.5 ml/well) and cultured for 1 day. The medium was then changed, and the cells were cultured for up to 48 hr. The HiBiT contents in the culture supernatant and cell lysate were determined using the Nano-Glo^®^ HiBiT Lytic Detection System (Promega). The signals in parental cells were subtracted from those in HiBiT clones, and the percent secretion per well was calculated. The parental cells were also transfected with either pBiT3.1-C-FGF1 or PM20D1, and HiBiT signals were measured and calculated as positive controls for secretion.

### Statistical analyses

The results are expressed as the mean ± S.E. values. Statistical significance was determined by Student’s *t* test for comparisons between two groups, one-way ANOVA followed by Tukey’s HSD (honestly significant difference) test for comparisons among three or more groups, and the chi-square test for comparisons of the muscle fiber cross-sectional area distribution. Statistical analyses were performed with EZR. A value of *P* < 0.05 was considered statistically significant.

## SUPPLEMENTARY MATERIALS



## Abbreviations

CC: Cancer cachexia
C22orf9: Chromosome 22 open reading frame 9
CM: Conditioned medium
NCM: Nonconditioned medium
PaCa: Pancreatic cancer
CRC: Colorectal cancer
GC: Gastric cancer
OSCC: Oral squamous cell carcinoma
TC: Tongue cancer
TA: Tibialis anterior
ER: Endoplasmic reticulum
