# Supplementary material for: The uncharacterized transcript KIAA0930 confers a cachexic phenotype on cancer cells
Source: Oncotarget. 2023 Jul 20;14:723–37. doi: 10.18632/oncotarget.28476 (PMC10360925; doi:10.18632/oncotarget.28476)
Supplement: Supplementary file 1 [file oncotarget-14-28476-s001.pdf]

The uncharacterized transcript *KIAA0930* confers a cachexic phenotype on cancer cells

SUPPLEMENTARY MATERIALS

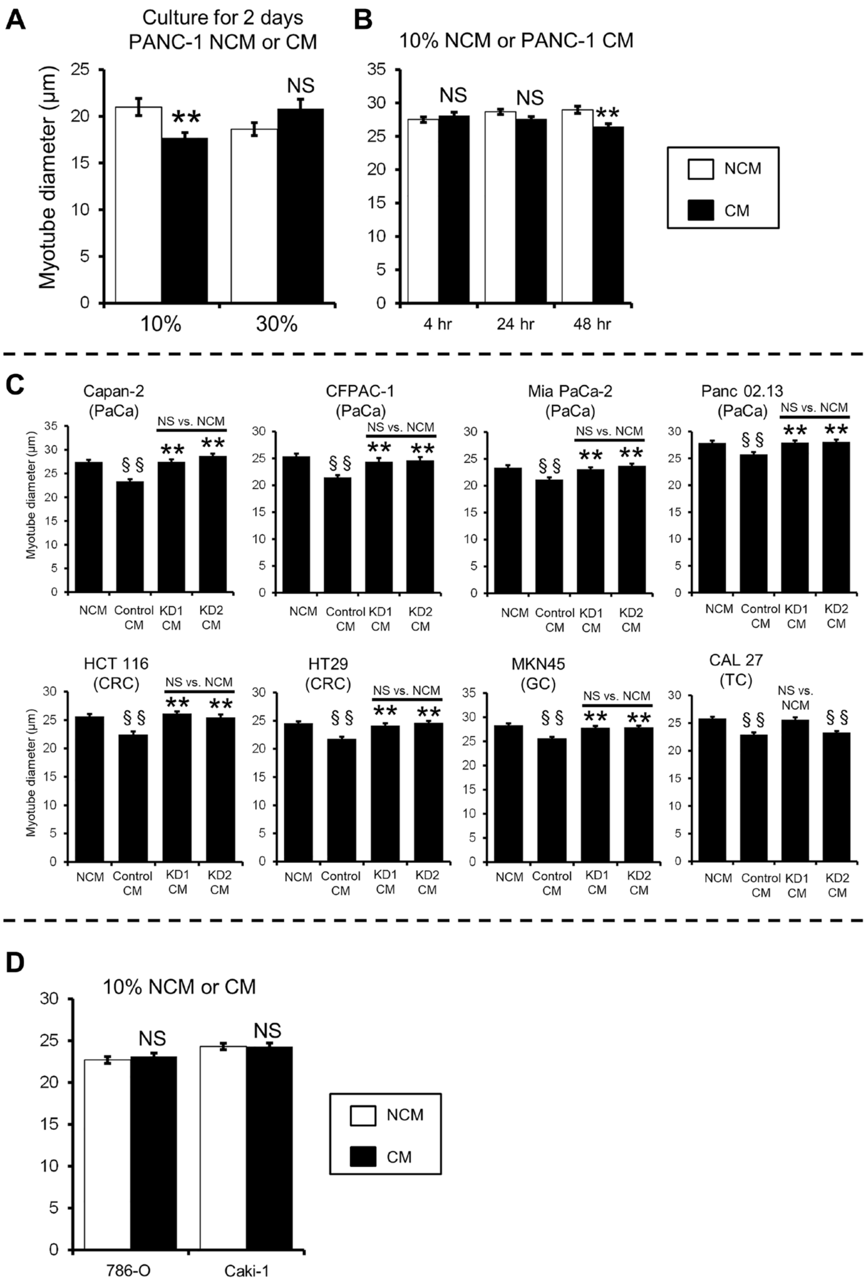

Supplementary Figure 1: The effects of conditioned medium from cancer cells on a myotube diameter in C2C12 cells. (**A** and **B**) Differentiated C2C12 myotubes were treated with 10 or 30% nonconditioned medium (NCM) or PANC-1 conditioned medium (CM) for up to 2 days. The myotubes were stained with MHC, and the diameters were measured. The data are shown as a representative of two independent experiments. (**C**) CM from *KIAA0930* knockdown cancer cells ameliorated myotube atrophy. (**D**) The myotubes were treated with 10% NCM or CM from 786-O or Caki-1 cells for 2 days, and the diameters were measured. The myotube diameter data are representative of at least three independent experiments and shown as a mean ± S.E. (n = 38–44 myotubes). $^{**}p < 0.01$ vs. Control CM; $^{\S\S}p < 0.01$ vs. NCM. Abbreviation: NS: not significant.

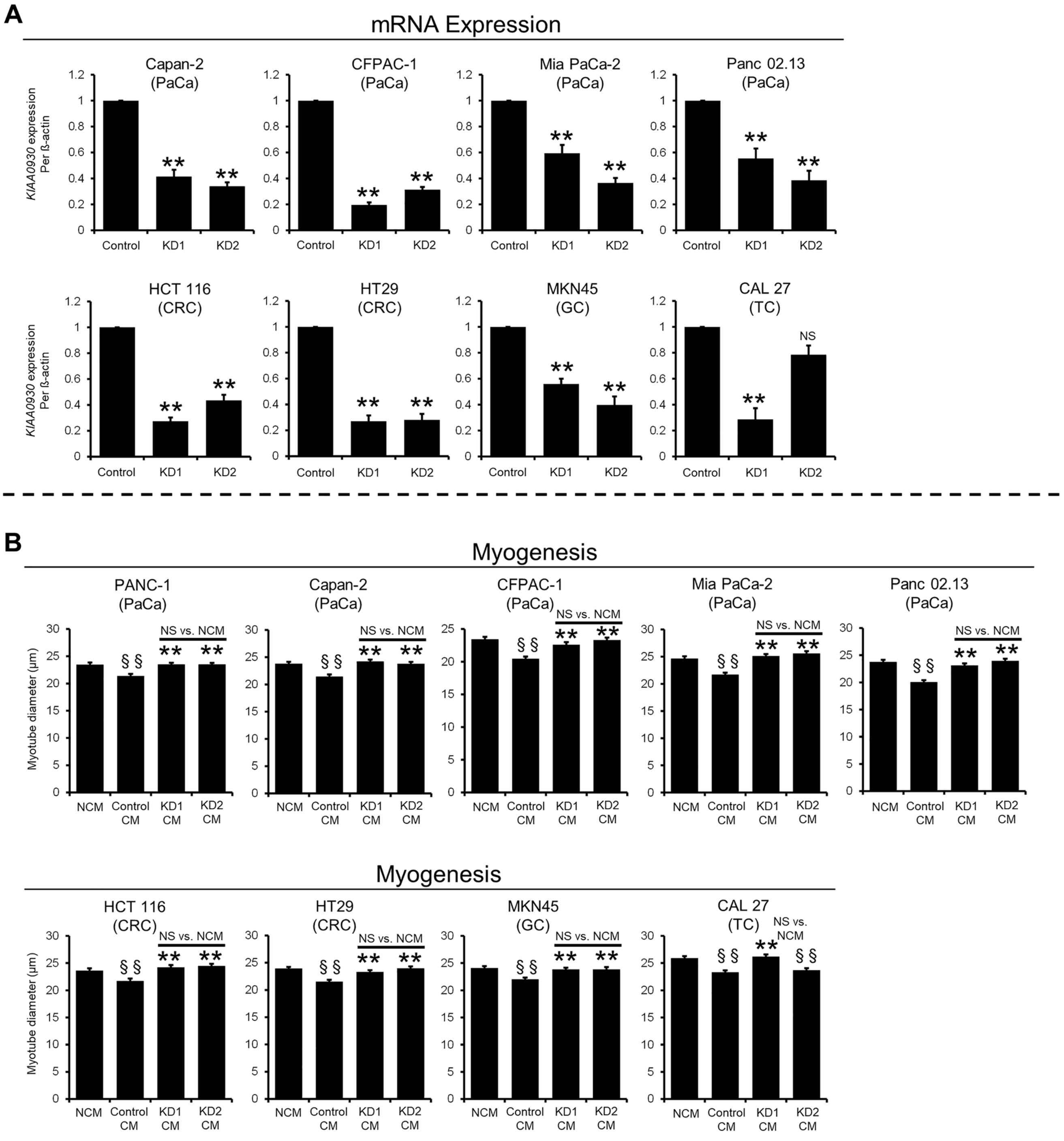

Supplementary Figure 2: *KIAA0930* mRNA expression in stable knockdown cells and the effects of conditioned medium from cancer cells on myogenesis in C2C12 cells. (**A**) mRNA expression of *KIAA0930* normalized by β-actin in control, KD1, and KD2 cancer cells. The data are shown as a mean ± S.E. from 3–11 independent experiments. $^{**}p < 0.01$ vs. Control. (**B**) The effect of conditioned medium (CM) from cancer cells on C2C12 myogenesis. Confluent C2C12 myoblasts were cultured with 10% NCM or CM from cancer cells (2% for CFPAC-1 and Panc 02.13 cells) for 5 days, and the diameter was measured. The myotube diameter data are shown as a representative of three independent experiments (mean ± S.E.; $n = 64$ myotubes). $^{**}p < 0.01$ vs. Control CM; $^{\S\S}p < 0.01$ vs. NCM. Abbreviation: NS: Not significant.

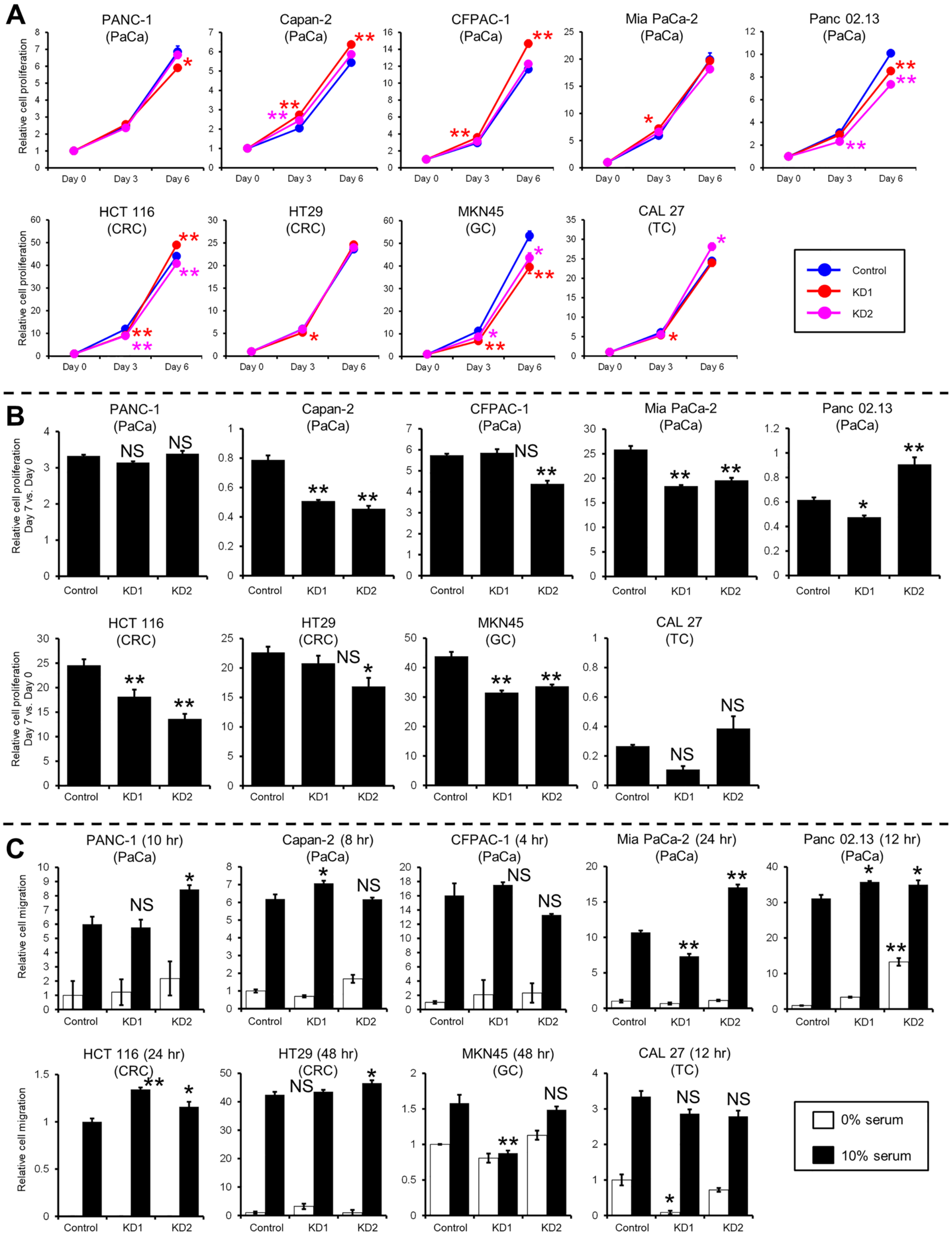

Supplementary Figure 3: The effects of *KIAA0930* knockdown on cell proliferation and migration. (**A**, **B**) Cell lines stably knocking down *KIAA0930* were seeded in a regular 96-well plate (A) or an ultralow attachement 96-well plate (B) and cultured for up to 7 days. Cell number was determined using CellTiter-Glo® 3D Cell Viability Assay kit. (**C**) The cell lines were seeded in culture insert with two chambers separated by a 500 µm wall and cultured until confluence. The insert was removed, cells were washed with serum-free medium, and cultured in serum-free or 10% serum-fed medium for the indicated time. Cells were then fixed, and migrated cell area was measured. The data are shown as mean ± S.E. ($n = 4$–6). $^{*}p < 0.05$; $^{**}p < 0.01$. Abbreviation: NS: Not significant vs. control cells.

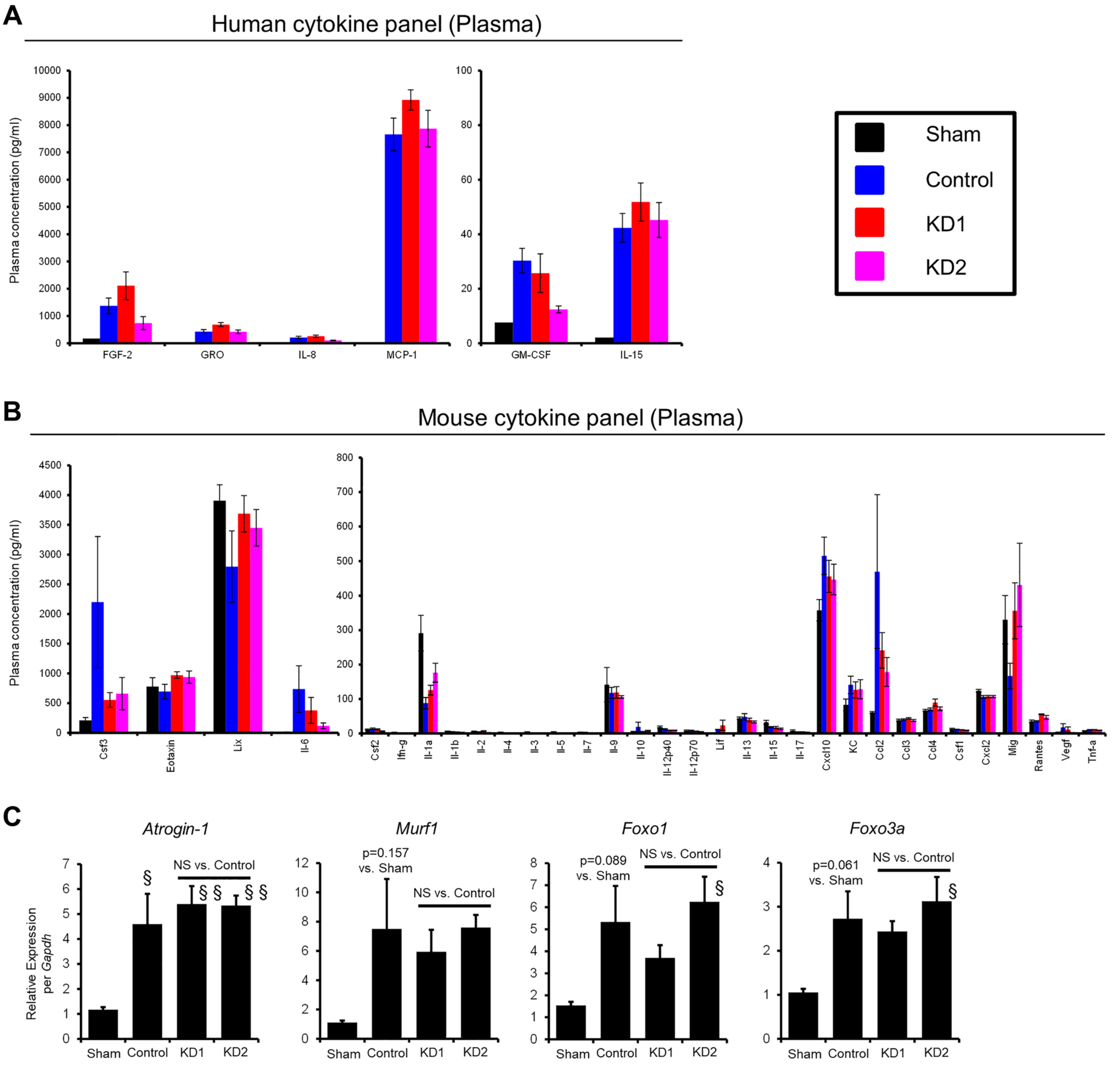

Supplementary Figure 4: Analyses of mouse TA muscle gene expression and tumor- or host-derived cytokines in the plasma of PANC-1 orthotopic xenograft model. (**A**) Human (tumor-derived) cytokine concentrations in plasma. FGF2, GRO, IL-8, MCP-1, GM-CSF, and IL-15 were detected, and other cytokines from human Cytokine/Chemokine 41 Plex panel were not detected. (**B**) Mouse (host-derived) cytokine concentrations in the plasma. (**C**) The gene expression in TA muscles normalized by *Gapdh*. The data are shown as Mean ± S.E. (n = 5–6 mice). §§$p < 0.01$; §$p < 0.05$ vs. Sham-operated group. Abbreviation: NS: Not significant.

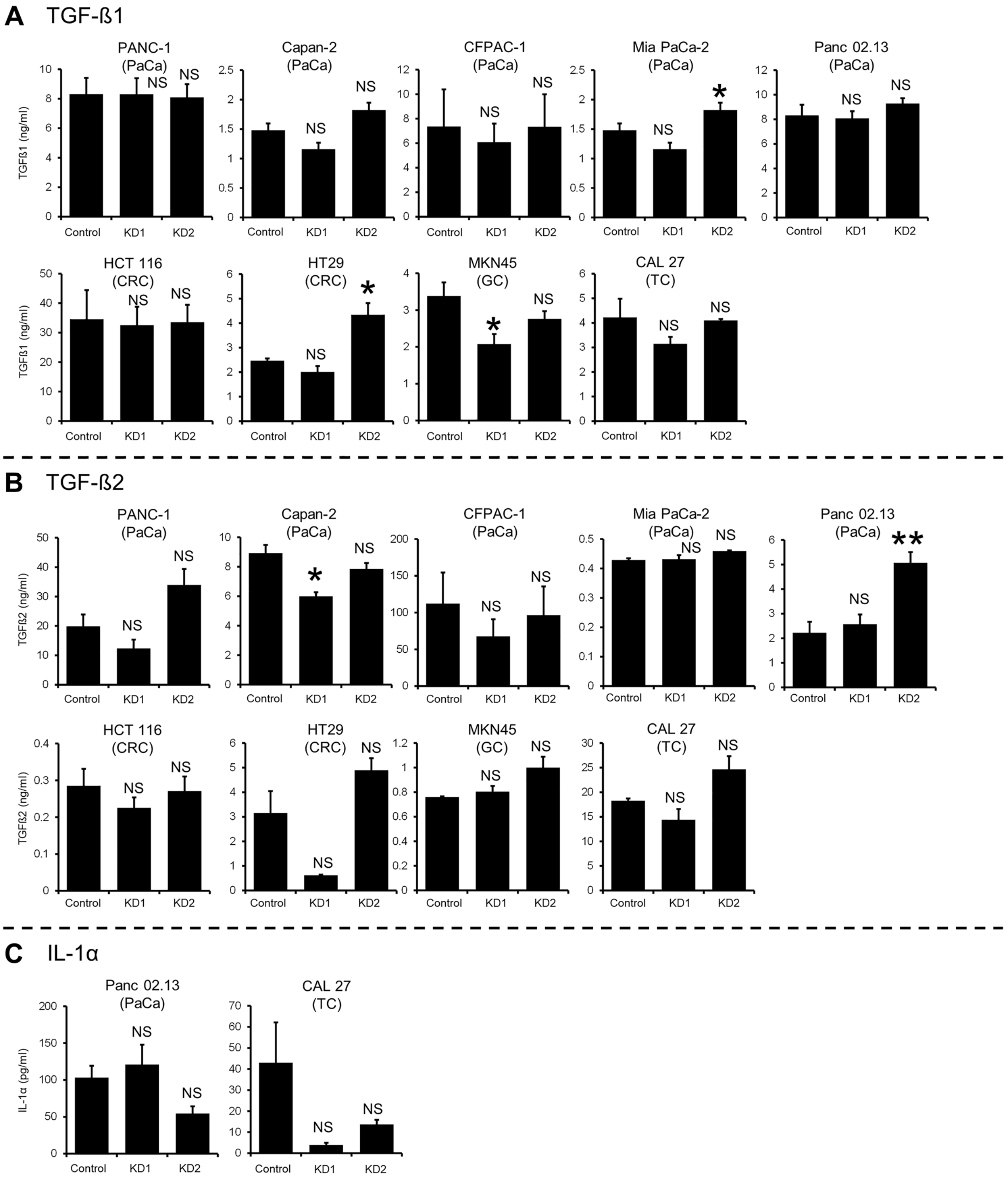

Less than detection limit (4 pg/ml): PANC-1, Capan-2, CFPAC-1, Mia PaCa-2, HCT 116, HT29, MKN45

Supplementary Figure 5: Cytokine/chemokine secretion from *KIAA0930* knockdown cancer cells. (**A**) transforming growth factor beta 1 (TGFß1); (**B**) TGFß2; (**C**) IL-1α, less than detection limit (4 pg/ml) in conditioned medium from PANC-1, Capan-2, CFPAC-1, Mia PaCa-2, HCT 116, HT29, and MKN45 cells; expression levels are shown. The data are shown as the mean ± S.E. from three independent experiments. $^{*}p < 0.05$; $^{**}p < 0.01$ vs. control. Abbreviation: NS: not significant.

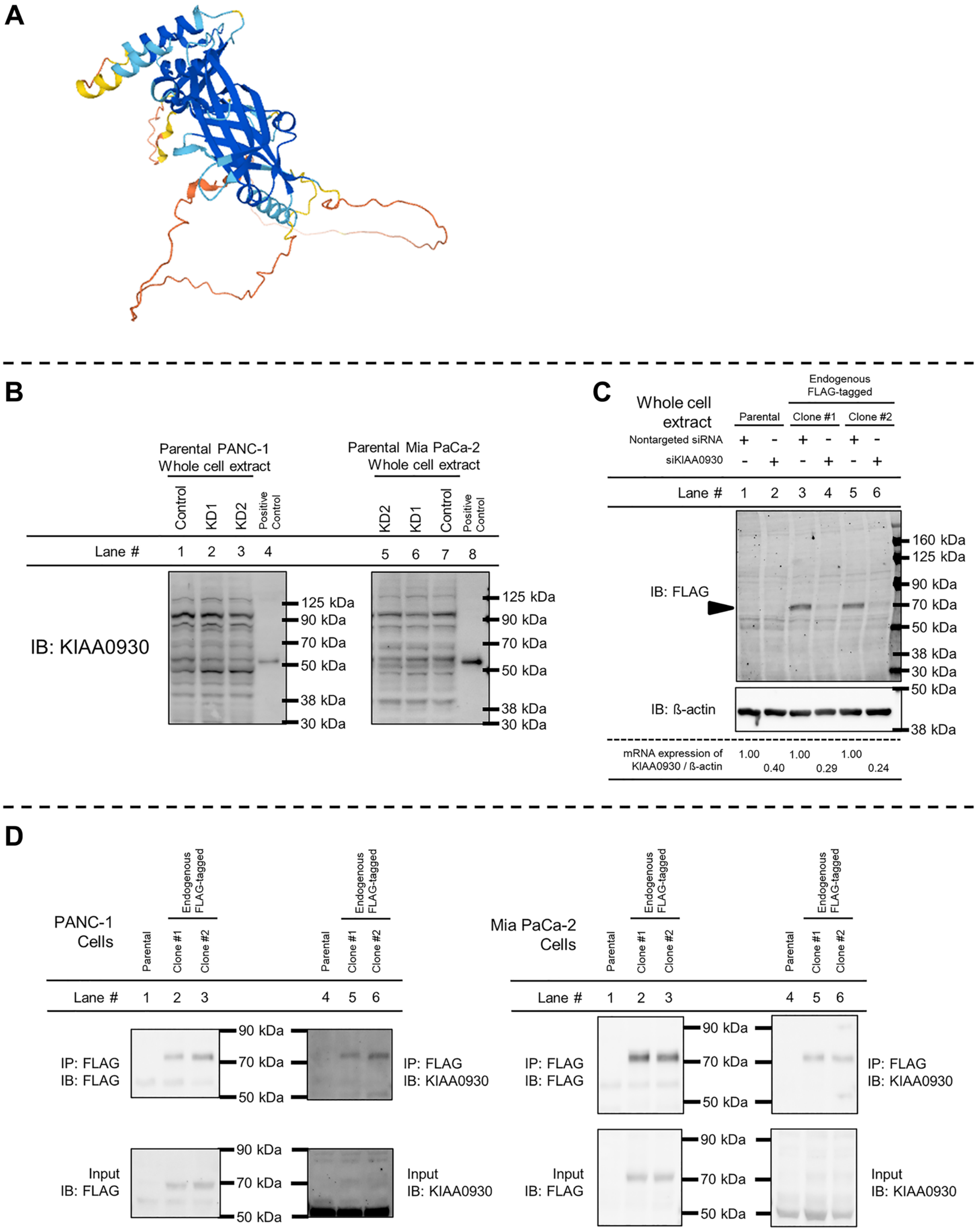

Supplementary Figure 6: Predicted three-dimensional structure of KIAA0930 protein and validation of endogenously FLAG-tagged PANC-1 and Mia PaCa-2 clones. (**A**) Predicted 3D structure of KIAA0930 protein with AlphaFold. (**B**) Representative Western Blotting figures for KIAA0930 in whole cell extracts from parental PANC-1 and Mia PaCa-2 cells. The KIAA0930 band was not able to be identified due to many nonspecific bands. (**C**) Parental Mia PaCa-2 cells and two FLAG clones were transfected with either nontargeted siRNA or siRNA targeted to *KIAA0930* (siKIAA0930), and whole cell extract and RNA were taken. The reduced mRNA expressions of KIAA0930 normalized to ß-actin in siKIAA0930-treated cells ensured sufficient knockdown of *KIAA0930*. Parental cells do not express FLAG whereas two FLAG clones express FLAG in nontargeted siRNA-treated cells. Knockdown of *KIAA0930* in these clones clearly decreased the FLAG signal. (**D**) Whole cell extracts from PANC-1 (Left) and Mia PaCa-2 (Right) were immunoprecipitated with anti-FLAG antibody, followed by elution with 3XFLAG peptide. Western Blotting was carried out for detection of FLAG and KIAA0930 proteins. These data indicate that the FLAG signal represents the KIAA0930 protein.

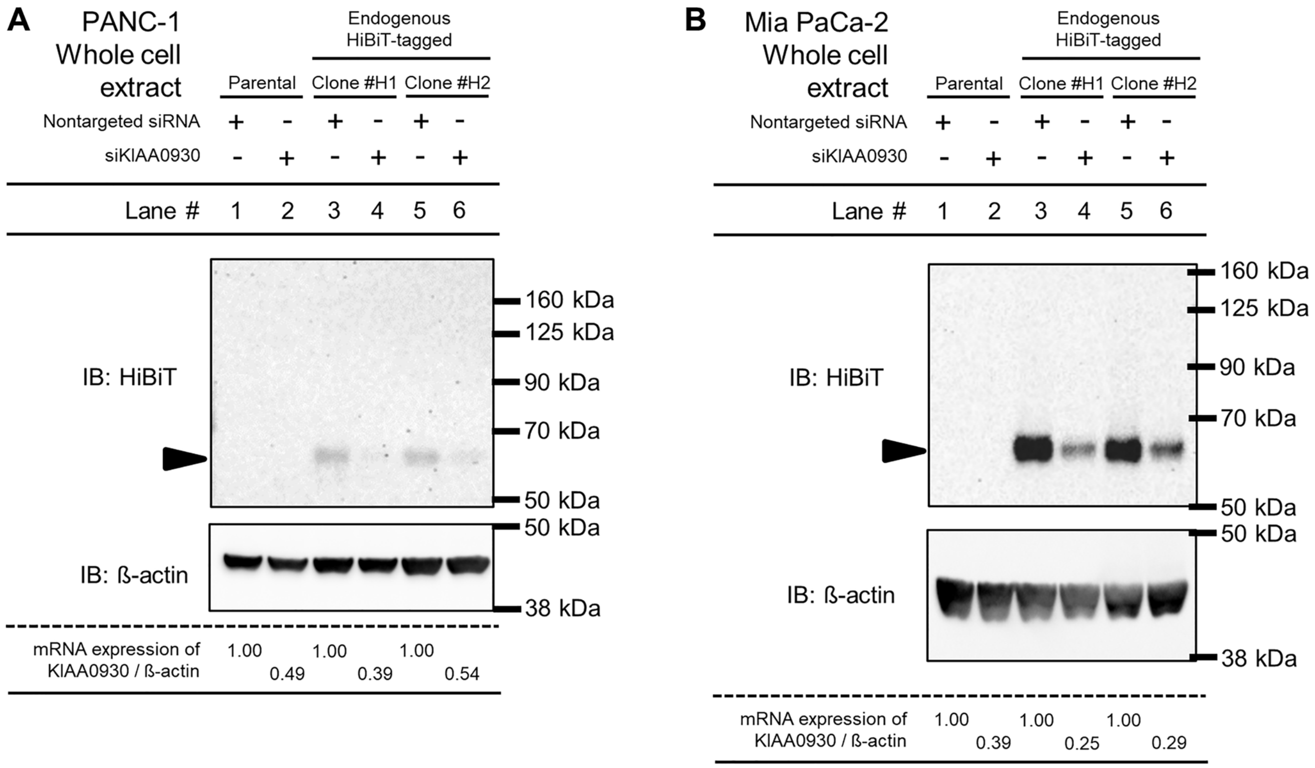

Supplementary Figure 7: Validation of endogenously HiBiT-tagged PANC-1 (**A**) and Mia PaCa-2 (**B**) clones. Cells were treated with either nontargeted siRNA or siKIAA0930 as mentioned in Supplementary Figure 6C, and HiBiT signals were detected using the Nano-Glo® HiBiT Blotting System.

Supplementary Table 1: *In vitro* myotube atrophy assay

	Myotube diameter (μm) Mean ± S.E. (n = 48–62)
NCM	27.501 ± 0.865
CM from nontargeted siRNA-treated PANC-1 cells	23.904 ± 0.855
CM from *C5orf13* knockdown PANC-1 cells	22.645 ± 1.111
CM from *C7orf42* knockdown PANC-1 cells	24.888 ± 1.195
CM from *C9orf16* knockdown PANC-1 cells	23.724 ± 0.919
CM from *C15orf48* knockdown PANC-1 cells	24.044 ± 0.966
CM from *C22orf9* knockdown PANC-1 cells	26.052 ± 0.899* (*$p < 0.05$ vs. NCM)

Supplementary Table 2: Relative light units in secretion assay

Cells/Fraction/Time	Relative light unit (reagent blank subtracted) Mean ± S.E. (n = 6)		
	Parental	Clone #H1	Clone #H2
PANC-1/CM/24 hr	173 ± 28	105 ± 13	189 ± 24
PANC-1/Cell lysate/24 hr	124 ± 4	11650 ± 352	15144 ± 282
PANC-1/CM/48 hr	249 ± 34	191 ± 19	225 ± 29
PANC-1/Cell lysate/48 hr	143 ± 10	14944 ± 106	17744 ± 182
Mia PaCa-2/CM/24 hr	482 ± 37	853 ± 118	612 ± 21
Mia PaCa-2/Cell lysate/24 hr	146 ± 6	28294 ± 406	23596 ± 249
Mia PaCa-2/CM/48 hr	590 ± 35	867 ± 85	538 ± 47
Mia PaCa-2/Cell lysate/48 hr	183 ± 9	45061 ± 1447	33747 ± 545

Supplementary Table 3: Relative light units in exosomal RNA transport assay

Conditioned medium to be treated with C2C12 mytobes	Relative light units in C2C12 myotubes (Reagent blank subtracted) Mean ± S.E. (n = 4)
Parental PANC-1 cells	106 ± 6
PANC-1 clone #H1 cells	103 ± 11
PANC-1 clone #H2 cells	113 ± 8
Parental Mia PaCa-2 cells	114 ± 7
Mia PaCa-2 clone #H1 cells	98 ± 7
Mia PaCa-2 clone #H1 cells	112 ± 3

Supplementary Table 4: shRNA sequences

shRNA	Sequence (palindromic oligos, <u>underline</u>: loop sequence)
Nontargeted (Control)	AAAAGTCCTCGACTAACACCGTTC<u>TTGGATCCAA</u>GAACGGTGTTAGTCGAGGAC
shKIAA0930 KD1	AAAAGACATTCACATCCATAAGAAG<u>TTGGATCCAA</u>CTTCTTATGGATGTGAATGTC
shKIAA0930 KD2	AAAAGCAACATGGAGTTTGTGCGCA<u>TTGGATCCAA</u>TGCGCACAAACTCCATGTTGC

Supplementary Table 5: Oligonucleotides for guideRNA targeted to *KIAA0930* gene

	Sequence (5′ -> 3′)
GuideRNA#1-top strand	CACCGACCGCATCGTCTTCTGGACT
GuideRNA#1-bottom strand	AAACAGTCCAGAAGACGATGCGGTC
GuideRNA#2-top strand	CACCGGCGGTGTGCACACGTGCTGA
GuideRNA#2-bottom strand	AAACTCAGCACGTGTGCACACCGCC

Supplementary Table 6: Primers for cloning of 5′- and 3′-homology arms (HOM) and for making HiBiT-tagged donor vector and HiBiT-tagged expression vectors

Primer name	Sequence (5′ -> 3′)
5′-HOM FWD	TCCCCGACCTGCAGCCCAGCTggccttctttcctccagccg
5′-HOM REV	CCGGAACCTCCTCCGCTCCCggtcatcaggatgggcttctgc
3′-HOM FWD	AGTTCTTCTGATTCGAACATCtgcggagcctgcgcagag
3′-HOM REV	TGGAGAGGACTTTCCAAGagccccaccccacatcagac
HiBiT_Donor FWD	5′Phos/GTGAGCGGCTGGCGGCTGTTCAAGAAGATTAGCgtttcaggaagcggagctactaacttcag
HiBiT_Donor REV	5′Phos/tccagaaccacctccaccg
ORF_pBiT3.1C FWD	CTATAGGGGCGATCGCAACAGGAGATCTGCCGCCGCGATCGCC
ORF_pBiT3.1C REV	CACACCGGAGCTCCCACCGCCGAGCGGCCGCGTACGCGT

Supplementary Table 7: Primers for real-time RT-qPCR

Targets	Sequence (5′ -> 3′)
Human KIAA0930 FWD	TCTTTCAGGGCTCCATCCGCTA
Human KIAA0930 REV	GCGCACAAACTCCATGTTGCTG
Human ß-Actin FWD	CACCATTGGCAATGAGCGGTTC
Human ß-Actin REV	AGGTCTTTGCGGATGTCCACGT
Mouse Atrogin-1 FWD	CTTCTCGACTGCCATCCTGGAT
Mouse Atrogin-1 REV	TCTTTTGGGCGATGCCACTCAG
Mouse Murf1 FWD	TACCAAGCCTGTGGTCATCCTG
Mouse Murf1 REV	ACGGAAACGACCTCCAGACATG
Mouse Foxo1 FWD	CTACGAGTGGATGGTGAAGAGC
Mouse Foxo1 REV	CCAGTTCCTTCATTCTGCACTCG
Mouse Foxo3a FWD	CCTACTTCAAGGATAAGGGCGAC
Mouse Foxo3a REV	GCCTTCATTCTGAACGCGCATG
Mouse Gapdh FWD	TCACCACCATGGAGAAGGC
Mouse Gapdh REV	GCTAAGCAGTTGGTGGTGCA
